# Validity and Reliability of Ventilatory and Blood Lactate Thresholds in Well-Trained Cyclists

**DOI:** 10.1371/journal.pone.0163389

**Published:** 2016-09-22

**Authors:** Jesús G. Pallarés, Ricardo Morán-Navarro, Juan Fernando Ortega, Valentín Emilio Fernández-Elías, Ricardo Mora-Rodriguez

**Affiliations:** 1 University of Castilla-La Mancha, Exercise Physiology Laboratory at Toledo, Toledo, Spain; 2 Human Performance and Sports Science Laboratory, Faculty of Sport Sciences, University of Murcia, Murcia, Spain; Norwegian University of Science and Technology, NORWAY

## Abstract

**Purpose:**

The purpose of this study was to determine, *i*) the reliability of blood lactate and ventilatory-based thresholds, *ii*) the lactate threshold that corresponds with each ventilatory threshold (VT_1_ and VT_2_) and with maximal lactate steady state test (MLSS) as a proxy of cycling performance.

**Methods:**

Fourteen aerobically-trained male cyclists ($\dot{\text{V}}{\text{O}}_{2\text{max}}$ 62.1±4.6 ml·kg^-1^·min^-1^) performed two graded exercise tests (50 W warm-up followed by 25 W·min^-1^) to exhaustion. Blood lactate, $\dot{\text{V}}{\text{O}}_{2}$ and $\dot{\text{V}}\text{C}{\text{O}}_{2}$ data were collected at every stage. Workloads at VT_1_ (rise in ${\dot{\text{V}}}_{\text{E}}/\dot{\text{V}}{\text{O}}_{2}$;) and VT_2_ (rise in ${\dot{\text{V}}}_{\text{E}}/\dot{\text{V}}\text{C}{\text{O}}_{2}$) were compared with workloads at lactate thresholds. Several continuous tests were needed to detect the MLSS workload. Agreement and differences among tests were assessed with ANOVA, ICC and Bland-Altman. Reliability of each test was evaluated using ICC, CV and Bland-Altman plots.

**Results:**

Workloads at lactate threshold (LT) and LT+2.0 mMol·L^-1^ matched the ones for VT_1_ and VT_2_, respectively (p = 0.147 and 0.539; r = 0.72 and 0.80; Bias = -13.6 and 2.8, respectively). Furthermore, workload at LT+0.5 mMol·L^-1^ coincided with MLSS workload (p = 0.449; r = 0.78; Bias = -4.5). Lactate threshold tests had high reliability (CV = 3.4–3.7%; r = 0.85–0.89; Bias = -2.1–3.0) except for D_MAX_ method (CV = 10.3%; r = 0.57; Bias = 15.4). Ventilatory thresholds show high reliability (CV = 1.6%–3.5%; r = 0.90–0.96; Bias = -1.8–2.9) except for RER = 1 and V-Slope (CV = 5.0–6.4%; r = 0.79; Bias = -5.6–12.4).

**Conclusions:**

Lactate threshold tests can be a valid and reliable alternative to ventilatory thresholds to identify the workloads at the transition from aerobic to anaerobic metabolism.

## Introduction

Maximal oxygen consumption [1], heart rate deflection [2], ventilatory/lactate thresholds [3,4] and maximum lactate steady state (MLSS) [5] are physiological evaluations related to endurance performance. Although all these tests predict, to some degree, endurance performance its accuracy, reproducibility and affordability varies. For instance, while maximal oxygen consumption could account for 91% of variability in marathon running performance, the velocity at lactate threshold explained 98% of the performance variability [6]. In turn, ventilatory threshold can accurately track the subtle improvements in endurance performance that elite cyclists obtain during an entire season [4]. Amann and co-workers [7] propose that ventilation, being under the influence of central and peripheral chemoreflex, is more sensitive and responsive to muscle hydrogen ion accumulation than the measures of blood lactate. Although workload at ventilatory threshold seems the more precise predictor of cycling endurance performance, often times the preferential use of ventilatory or lactate thresholds depends on equipment availability and ease at data interpretation.

Physiological testing is not only useful to predict performance, but to design successful training programs. Endurance training geared to enhance performance is more efficient when workloads are individually prescribed relative to the aerobic-anaerobic transition workload compared to estimated workload by reference to a maximum (e.g. percentages of maximal heart rate or about maximal aerobic power) [8]. Identification of anaerobic threshold using indirect calorimetry (ventilatory threshold) during a graded exercise test is habitual in research laboratories, professional teams and high performance national centers [4]. However, reliable metabolic carts are expensive (approx. 30,000 $) and thus are a limited resource for many trainers and athletes. Evaluation of anaerobic threshold by capillary blood lactate (CBL) is cheaper and thus often chosen as an alternative method. However, the validity [7] and reliability [9,10] of aerobic-anaerobic CBL detection remains controversial.

Thus, performance assessment and training prescription is often based on blood lactate concentration changes during a graded exercise test (GXT). Some authors define “lactate threshold” as the workrate beyond which, blood lactate concentration rises above resting level [11]. Other authors sustain that the workload that elicits a blood lactate concentration 1 mMol·L^-1^ above resting levels is better related to endurance performance [12]. To avoid the bias of visual identification of lactate threshold, curve fitting procedures have been used such as the D_MAX_ method [13]. Other investigators have proposed that identification of the workload that elicit a fixed lactate level of 4 mM can predict endurance performance [14]. Finding the highest workload that however does not results in increasing blood lactate concentration is the aim of the MLSS test [15]. Although blood lactate tests to predict performance proliferate, there has been few studies comparing the validity of these in comparison to ventilatory thresholds (i.e., VT_1_ and VT_2_).

Some investigations have studied the reliability of VT_1_, VT_2_ and lactate thresholds (LT) using a graded exercise testing protocol [7, 16]. However, to our knowledge, nobody has investigated the agreement and differences between blood lactate and ventilatory thresholds during the same incremental test. Thus, the purpose of this study was to assess the validity and reliability of critical workloads found using lactate thresholds or lactate levels in comparison to the more accepted ventilatory determined thresholds.

## Materials and Methods

### Subjects

Fourteen trained men cyclists volunteered to participate in this study (age 26.7 ± 8.2 yr, body mass 70.3 ± 4.9 kg, height 173.7 ± 4.2 cm, body fat 12.5 ± 3.0%, $\dot{\text{V}}{\text{O}}_{2\text{max}}$ 62.1 ± 4.6 ml·kg·min^-1^, endurance training experience 10.9 ± 4.9 yr). No physical limitations or musculoskeletal injuries that could affect training were reported. Cyclist underwent a complete medical examination (including ECG) that showed all were in good health. This study was conducted during the period from January 2014 to July 2015. The study, which was conducted according to the declaration of Helsinki, was approved by the Bioethics Commission of the University of Murcia. Written informed consent was obtained from all subjects prior to participation.

### Experimental Design

Following a familiarization GXT, participants rested for 48 hours to ensure adequate recovery. Participants visited the lab 5–7 times separated by 2–5 days. In the first two sessions, cyclists performed two identical GXT to establish the average power output (W) associated to 14 different aerobic-anaerobic events based on ventilatory gas exchange and CBL. Thereafter, participants visited the lab 3–5 more times to determine the workload associated with the maximal lactate steady state (MLSS). All trials were performed between 16:00 h—19:00 h to control the circadian rhythms effects [17], under similar environmental conditions (21–24°C and 45–55% relative humidity). In all trials subjects were ventilated at a wind velocity of 2.55 m·s^-1^ with a fan positioned 1.5 m from the subject’s chest. A training protocol was established with the objective of maintaining physical performance individualized to each cyclist for the entire investigation period (5–6 weeks), always keeping 24 hour of full recovery prior to each assessment session. This training program consisted in cycling sessions of 90 minutes every 48 hours at the individual intensity of nVT_1_ interspersed with efforts of 5–7 min at 90–95% intensity of VT_2_ each 20 min.

### Maximal graded exercise tests

Participants performed all the experimental trials on the same cycle ergometer (Ergoselect 200, Ergoline, Germany). Immediately following a standardized warm-up of 10 min at 50 W, all participants performed a ramp protocol with increments of 25 W·min^-1^ until exhaustion. During GXT participants were monitored by standard 12 lead ECG (Quark T12, Cosmed, Italy), Oxygen consumption ($\dot{\text{V}}{\text{O}}_{2}$) and carbon dioxide production ($\dot{\text{V}}\text{C}{\text{O}}_{2}$) were recorded using breath-by-breath indirect calorimetry (Quark B^2^, Cosmed, Italy). Familiarization GXT fulfilled three objectives: a) discard cardiac defects or diseases in any of the participants, b) to minimize the bias of progressive learning on test reliability and c) to discard any participant $\dot{\text{V}}{\text{O}}_{2\text{max}}$ lower than 55.0 ml·kg^-1^·min^-1^.

Both experimental maximal GXT with 15 min warm-up divided in three 5-min steady state stages at 45%, 55%, and 65% of the peak power output (PPO), being the three intensities below the second ventilatory threshold (VT_2_). After 10 minutes of passive recovery in which each participant ingested 200–250 ml of water to ensure adequate hydration status, a sample of capillary blood from the finger was obtained to assess CBL (Lactate Pro, Arkray, Japan). Following, participants performed the GXT according to a modification of the protocol described by [4]. Initial workload was set at 50 W, with increments of 25 W·min^-1^, requiring at all times a cadence between 80–85 rpm.

Heart rate was continuously monitored (RS400, Polar, Finland), gas exchange was recorded breath by breath using indirect calorimetry and capillary blood samples were obtained and analyzed every 2 min (i.e., each 50 W increments). Each participant indicated their rate of perceived exertion every two minutes using the Borg Scale 6–20, where 6 is defined as an effort "very very light" and one 20 "Maximum, strenuous" effort [18]. Capillary blood lactate analyzer and indirect calorimetry devices were calibrated before each test. In order to avoid the local acidosis that could impair the attainment of maximum cardiorespiratory performance, and according to the subjects’ maximal PPO in the GXT_PRE_ (i.e., 375-425W), starting at 50 W, the workload was progressively increased by 25 W·min^-1^ that ensure that testing duration was not excessively long (i.e., 13.5–15.0 min). This protocol also allowed collecting between 7 to 9 capillary blood samples before exhaustion to be used in the CBL data analysis.

### Maximal lactate steady state test

**Fig 1:**
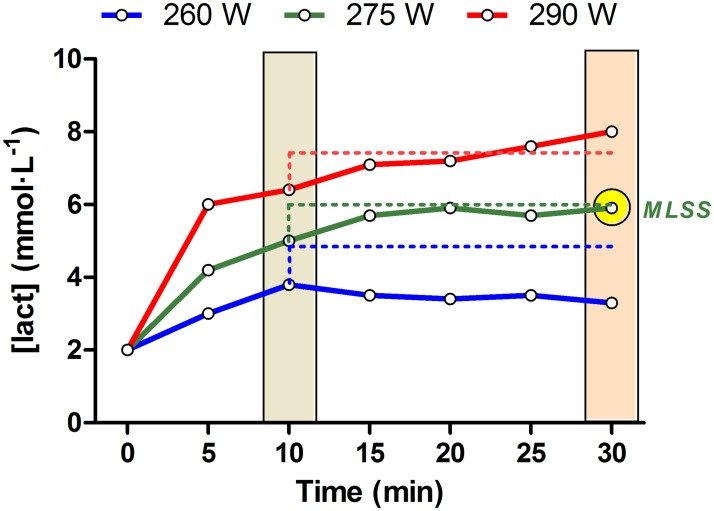
Example of determination of maximal lactate steady state.

Several 30 min constant workloads pedaling were performed to identify the highest workload (i.e. W) which elicited an increment in BLC less than 1 mMol between 10 and 30 min of exercise [5,19]. After 7 days from the second GXT, all participants performed the first MLSS trial at the individual workload associated to their respective lactate threshold (LT) determined during the GXT. Depending on the result of the first MLSS test, the workload of the second and following MLSS tests increased or decreased 0.2 W·Kg^-1^ (~ 15 W), until criteria was fulfilled. Between 3 and 5 tests were necessary to determine the workload (i.e. W) associated MLSS for each cyclist (Fig 1).

### VO_2_max and ventilatory thresholds determinations during the GXT

**Fig 2:**
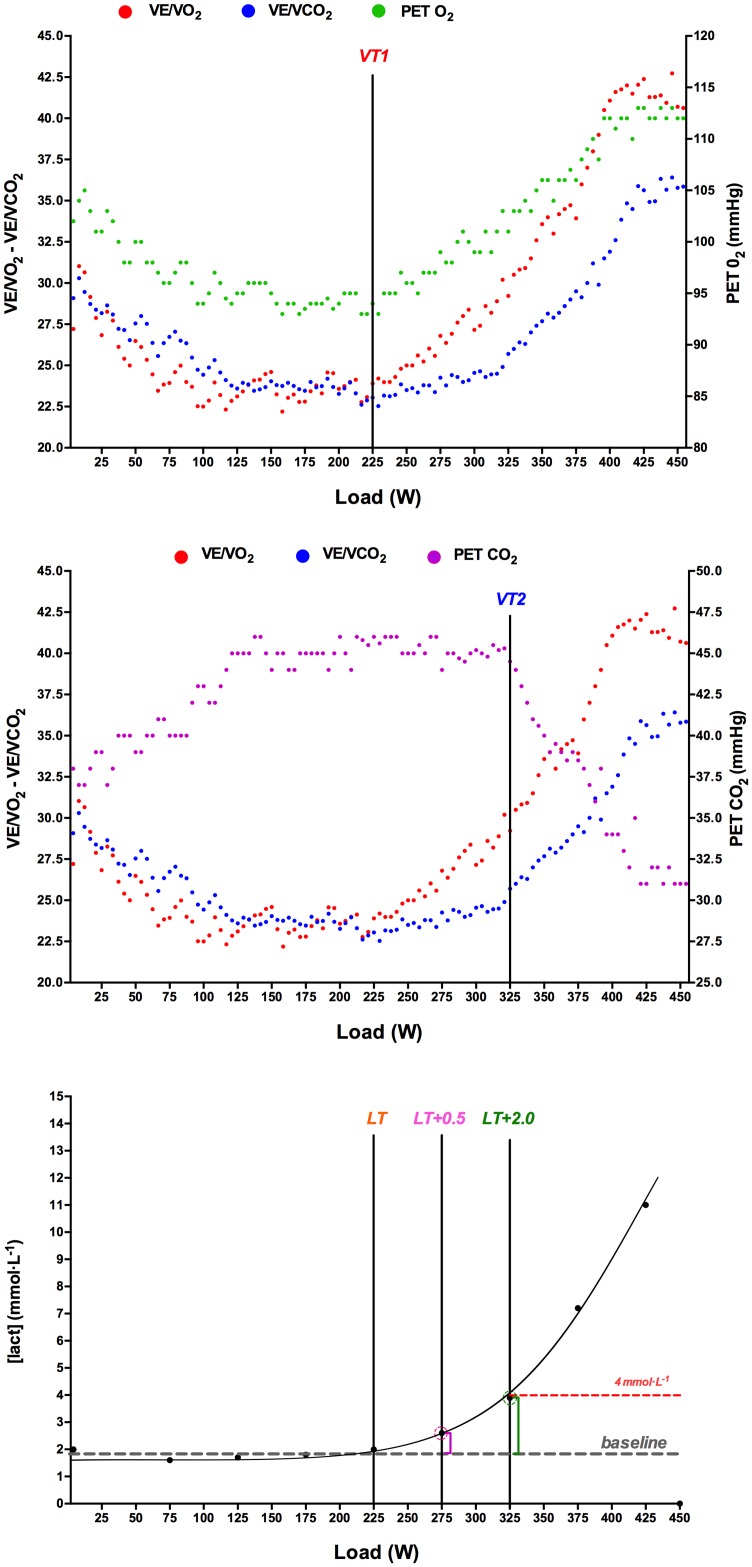
Example of determination of lactate threshold (LT) as well as first (VT_1_) and second ventilatory thresholds (VT_2_) in one test. Each gas-exchange data point corresponds to a 10-s interval. ${\dot{\text{V}}}_{\text{E}}/\dot{\text{V}}{\text{O}}_{2}$, ventilatory equivalent for oxygen; ${\dot{\text{V}}}_{\text{E}}/\dot{\text{V}}\text{C}{\text{O}}_{2}$, ventilatory equivalent for carbon dioxide; P_ET_CO_2_, end-tidal pressure of oxygen; end-tidal pressure of carbon dioxide (P_ET_CO_2_).

VT_1_ was determined using the criteria of an increase in both ventilatory equivalent of oxygen (${\dot{\text{V}}}_{\text{E}}/\dot{\text{V}}{\text{O}}_{2}$) and end-tidal pressure of oxygen (P_ET_O_2_) with no concomitant increase in ventilatory equivalent of carbon dioxide (${\dot{\text{V}}}_{\text{E}}/\dot{\text{V}}\text{C}{\text{O}}_{2}$). VT_2_ was determined using the criteria of an increase in both the ${\dot{\text{V}}}_{\text{E}}/\dot{\text{V}}{\text{O}}_{2}$ and ${\dot{\text{V}}}_{\text{E}}/\dot{\text{V}}\text{C}{\text{O}}_{2}$ and a decrease in P_ET_CO_2_ [4]. Maximal oxygen uptake (i.e., $\dot{\text{V}}{\text{O}}_{2\text{max}}$) was defined as the highest plateau (two successive maximal readings within 0.15 L/min) reached. V-slope workload was identified in that intensity of exercise which, in a plot of the minute production of CO_2_ over the minute utilization of oxygen ($\dot{\text{V}}{\text{O}}_{2}$), shows an increase in the slope above 1.0 [20, 21]. The workload associated with a respiratory exchange ratio equal to unity was defined as RER = 1.00 (Fig 2).

### Capillary blood lactate thresholds during the GXT

Lactate Threshold (LT) was determined by examining the lactate concentration-workload relationship ([Lact]_blood_/W) during the GXT as the highest workload not associated with a rise in lactate concentration above baseline [9]. Baseline lactate concentration was the average during the initial stages with values 0.5 mMol·L^-1^ above rest state. This always occurred just before the curvilinear increase in blood lactate observed at subsequent exercise intensities [4,22].

Lactate Threshold + 1.0 mM·L^-1^ (LT+1.0) represents the workload (W) which causes an increase of 1 mM·L^-1^ above baseline measurements [22]. As a novel contribution of this study, five new lactate thresholds were established following the same criterion as detailed to determine the LT+1.0 mM·L^-1^ (i.e. concentrations above baseline). Accordingly, the following thresholds were established: LT+0.5, LT+1.5, LT+2.0, LT+2.5, and LT+3.0 mM·L^-1^, carrying out an interpolation results for each of the concentrations proposed (Fig 2).

D_MAX_ threshold was determined by plotting the lactate response to exercise intensity in a third-order polynomial regression curve. The D_MAX_ was defined as the point on the regression curve that yields the maximal distance to the straight line formed by the two end points of the curve [13].

Onset of blood lactate accumulation (OBLA_4mM_) was defined as the exercise intensity (W) identified by interpolation across the 4 mM·L^-1^ point in the plot of [Lact]_blood_ during incremental exercise [14]. Two independent observers detected all ventilatory and lactate thresholds following the criteria previously described. If they did not agree, the opinion of a third investigator was sought [23].

### Body composition

Fat-free mass and fat mass were assessed by X-ray absorptiometry dual energy (DXA) (Hologic Discovery, Hologic Corp., Waltham, MA, USA). Participant’s height and weight were assessed in a stadiometer (Seca 202, Seca Ltd., Hamburg, Germany) and body mass index was calculated.

### Statistical analysis

Standard statistical methods were used for the calculation of means, standard deviations (SD) and 95% confidence interval. The validity against the three Gold Standard methods (i.e., VT_1_, MLSS and VT_2_) was assessed using one-way repeated measures ANOVA followed by pairwise comparisons (Bonferroni’s adjustment), intraclass correlation coefficient (ICC) and Bland–Altman plots [24]. The reliability of ventilatory and lactate determinations was assessed using coefficients of variation (CV), ICC and Bland–Altman plots. The size of the correlations was evaluated as follows; r < 0.7 low; 0.7 ≤ r < 0.9 moderate and r ≥ 0.9 high [25]. Analyses were performed using GraphPad Prism 6.0 (GraphPad Software, Inc., CA, USA) and SPSS software version 19.0 (SPSS, Chicago, IL).

## Results

### Validity

**Fig 3:**
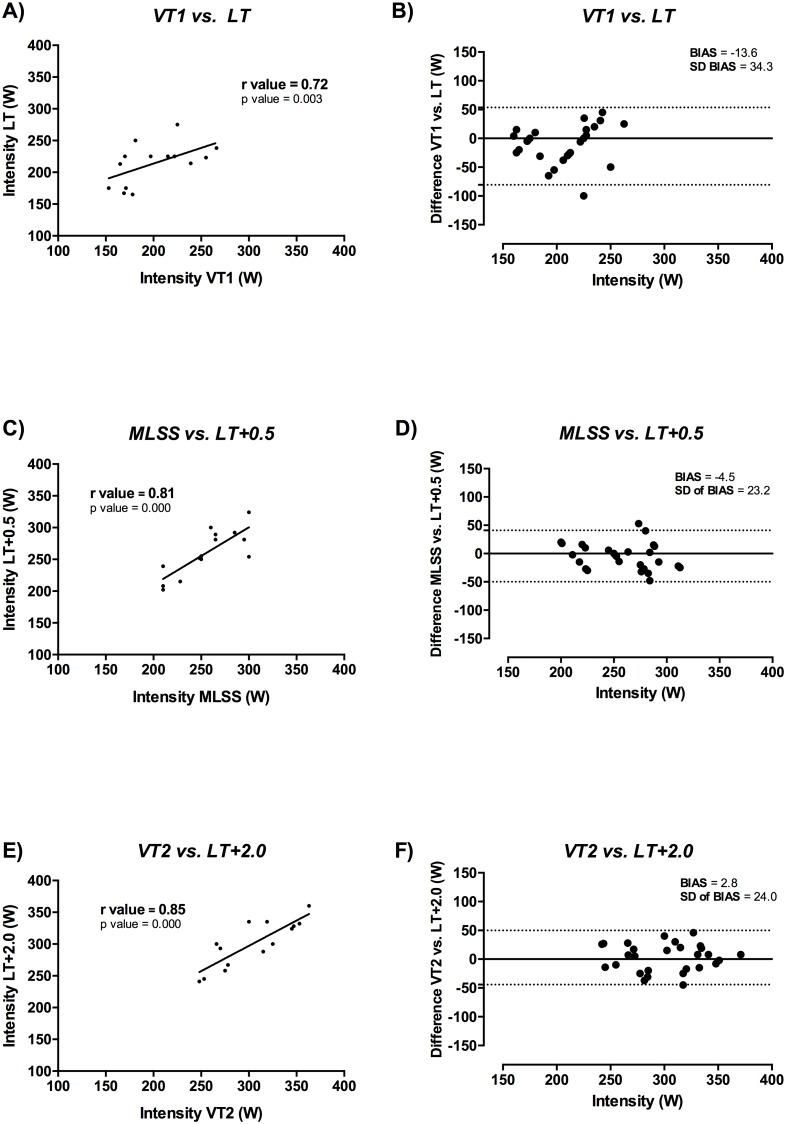
CCI and Bland Altman results.

VT_1_ workload (200 ± 36 W) was different to the workload for the rest of the CBL thresholds except for the LT threshold (214 ± 33 W, p = 0.147; Table 1). Accordingly, the higher correlation coefficient between VT_1_ and CBL was obtained with LT (r = 0.72, p < 0.05; Fig 3A). Likewise, Bland-Altman analysis (Table 1) showed the highest agreement (i.e., lower bias) for the LT method (-13.6 ± 34.3; Fig 3B). The workload at MLSS (255 ± 32 W) was different from the workload obtained with the rest of the thresholds except for RER = 1 (259 ± 36 W, p = 0.750), LT +0.5 (260 ± 36 W, p = 0.449) and D_MAX_ (257 ± 40 W, p = 0.830) (Table 1). Meanwhile, LT+0.5—LT+3.0 and OBLA_4mMol_ had the higher coefficient of correlation against MLSS (r > 0.78, p < 0.05 in both cases, Fig 3C). Bland-Altman analysis (Table 1 and Fig 3D) revealed less bias in LT+0.5 (-4.5 ± 23.2), D_MAX_ (-1.8 ± 38.1) and RER = 1 (-3.8 ± 45.5).

**Table 1:** Validity results of used methods. Comparison of workload at VT_1_, MLSS and VT_2_ against workloads.

			VT_1_	MLSS	VT_2_	RER = 1	V-SLOPE	LT	LT+0.5	LT+1.0	LT+1.5	LT+2.0	LT +2.5	LT +3.0	DMAX	OBLA_4mM_
	Workload (W; Mean ± SD)		200±36	255±32	304±39	259±36	235±32	214±33	260±36	272±36	288±37	300±37	311±38	320±39	257±40	304±40
[Lact] (mmol·L^-1^; Mean ± SD)			1.1±0.4	4.5±0.9	4.2±1.0	2.8±1.3	2.1±0.6	1.6±0.3	2.1±0.3	2.6±0.3	3.1±0.3	3.6±0.3	4.1±0.3	4.6±0.3	2.8±1.4	4.0±0.0
	Differences (W)			55	104	59	35	14	60	72	88	100	111	120	57	104
First Ventilatory Threshold (**VT**_**1**_) 200±36 W	**Mean Differences**—p value			0.000	0.000	0.000	0.000	0.147	0.000	0.000	0.000	0.000	0.000	0.000	0.000	0.000
	**ICC**	r value		0.62	0.73	0.27	0.72	0.72	0.68	0.65	0.66	0.66	0.42	0.44	0.35	0.43
		p value		0.016	0.002	0.346	0.003	0.003	0.008	0.010	0.010	0.01	0.02	0.02	0.215	0.125
	**Bland Altman**															
	Bias			-55.2	-102.7	-59.0	-34.8	-13.6	-59.7	-72.4	-87.4	-99.9	-110.9	-119.6	-61.6	-104.00
	SD of Bias			30.3	28.1	45.8	28.4	34.3	40.0	39.0	39.9	39.7	40.5	40.0	53.1	40.09
	Differences (W)		55		49	4	-20	-41	5	17	33	45	66	65	2	49
Maximal Lactate Steady State (**MLSS**) 255±32 W	**Mean Differences**—p value		0.000		0.000	0.750	0.006	0.000	0.449	0.009	0.000	0.000	0.000	0.000	0.830	0.000
	**ICC**	r value	0.61		0.85	0.17	0.70	0.76	0.78	0.80	0.81	0.82	0.83	0.84	0.56	0.82
		p value	0.001		0.000	0.397	0.000	0.000	0.000	0.000	0.000	0.000	0.000	0.000	0.002	0.000
	**Bland Altman**															
	Bias		55.2		-47.5	-3.8	19.0	41.6	-4.5	-17.1	-32.1	-44.7	-55.7	-64.4	-1.8	-48.93
	SD of Bias		30.3		19.9	45.5	25.3	22.4	23.2	21.7	22.0	21.6	21.7	21.0	38.1	21.64
	Differences (W)		-104	-49		-43	-69	-90	-44	-32	-16	-4	7	16	-47	0
Second Ventilatory Threshold (**VT**_**2**_) 304±39 W	**Mean Differences**—p value		0.000	0.000		0.003	0.000	0.000	0.000	0.000	0.015	0.539	0.250	0.018	0.000	0.965
	**ICC**	r value	0.85	0.85		0.46	0.82	0.75	0.80	0.80	0.80	0.80	0.79	0.80	0.62	0.74
		p value	0.000	0.000		0.013	0.000	0.000	0.000	0.000	0.000	0.000	0.000	0.000	0.000	0.000
	**Bland Altman**															
	Bias		-102.7	47.5		43.7	67.9	89.1	43.0	30.4	15.4	2.8	-8.2	-16.9	41.1	-1.2
	SD of Bias		28.1	19.9		38.5	22.2	25.0	23.5	23.2	23.7	24.0	25.2	24.2	39.6	28.5

*VT*_*1*_ First ventilatory threshold, *MLSS* Maximal lactate steady state, *VT*_*2*_ Secondary ventilatory threshold, *RER = 1* Respiratory exchange ratio = 1, *LT* Lactate threshold, *LT+0*.*5*,*+1*.*0*,*+1*.*5*,*+2*.*0*,*+2*.*5*,*+3*.*0* Concentrations above lactate threshold, *D*_*MAX*_ Maximum distance between the slope of a polynomial and the line connecting both ends, *OBLA*_*4mMol*_ Onset blood lactate accumulation 4 mM

VT_2_ workload (304 ± 39 W) was similar to LT +2.0 (300 ± 37 W, p = 0.539, Fig 3), LT+2.5 (311 ± 38 W, p = 0.250) and OBLA_4mM_ (304 ± 40 W, p = 0.965) (Table 1). The highest correlation coefficient between VT_2_ and CBL was with LT—LT+3.0 (r > 0.79, p < 0.05; Table 1, Fig 3E). Lactic determinations with less bias in the Bland-Altman test were LT +2.0 (2.8 ± 24.0; Fig 3F) and OBLA_4mM_ (-1.2 ± 28.5) (S1 File).

### Reliability

Intra-subject reliability (GXT I vs. GXT II) of both gold standard thresholds (VT_1_ and VT_2_) revealed low CV (3.6% and 2.1%), high ICC (r = 0.95–0.96) and low Bland-Altman bias (-2.9 ± 13.3 and -2.7 ± 11.4) suggesting high level of agreement. Similarly, the intra-subject reliability associated to lactate thresholds (LT—LT+3.0) and the OBLA_4mMol_ were high (CV = 3.0%-3.7%; r = 0.85–0.88; p < 0.000; Bias = 1.3 ± 18.8–2.9 ± 19.0; Table 2). However, D_MAX_ and RER = 1 had higher CV (10.3–6.4%), lower ICC (r = 0.57–0.79) and higher Bland-Altman bias (15.4 ± 42.9 and -12.4 ± 24.7) suggesting poor reliability (S1 File).

**Table 2:** Reliability of lactate and ventilatory tests. CV, ICC and Bland-Altman results.

Workload (W; Mean ± SD)	$\dot{\text{V}}{\text{O}}_{2\text{max}}$ 388 ± 32	VT_1_ 200 ± 36	VT_2_ 304 ± 39	RER = 1 259 ± 36	V-Slope 235 ± 32	LT 214 ± 33	LT+0.5 260 ± 36	LT+1.0 272 ± 36	LT+1.5 288 ± 37	LT+2.0 300 ± 37	LT+2.5 311 ± 38	LT+3.0 320 ± 39	D_MAX_ 257 ± 40	OBLA_4mM_ 304 ± 40
**CV (%)**	1.6%	3.5%	2.1%	6.4%	5.0%	3.7%	3.7%	3.4%	3.4%	3.4%	3.4%	3.0%	10.3%	3.7%
**ICC**														
**r value**	0.90	0.95	0.96	0.79	0.79	0.85	0.89	0.88	0.88	0.88	0.87	0.89	0.57	0.85
**p value**	0.000	0.000	0.000	0.001	0.001	0.000	0.000	0.000	0.000	0.000	0.000	0.000	0.031	0.000
**Bland Altman**														
**Bias**	-1.8	-2.9	-2.7	-12.4	-5.6	-2.1	1.9	2.0	1.3	2.9	2.0	3.0	15.4	1.8
**SD of Bias**	15.4	13.3	11.4	24.9	22.3	18.7	18.1	17.9	18.8	19.0	20.2	19.1	42.7	22.6

*$\dot{\text{V}}\text{O}2_{\text{max}}$* Maximal oxygen consumption, *VT*_*1*_ First ventilatory threshold, *MLSS* Maximal lactate steady state, *VT*_*2*_ Secondary ventilatory threshold, *RER = 1* Respiratory exchange ratio = 1, *LT* Lactate threshold, *LT+0*.*5*,*+1*.*0*,*+1*.*5*,*+2*.*0*,*+2*.*5*,*+3*.*0* Concentrations above lactate threshold, *D*_*MAX*_ Maximum distance between the slope of a polynomial and the line connecting both ends, *OBLA*_*4mMol*_ Onset blood lactate accumulation 4 mM

## Discussion

The first aim of this study was to identify during a graded exercise test (25 W·min^-1^), which blood lactate concentration threshold (LT, LT+0.5, LT+1, LT+1.5, LT+2.0, LT+2.5, LT+3.0 mM·L^-1^, D_MAX_ or OBLA_4mM_) better matched the workload at ventilatory thresholds (VT_1_ and VT_2_). The ultimate goal is to provide coaches and athletes with a valid alternative test to obtain performance workloads without the need of using indirect calorimetry (less affordable technology). In addition, we tested the reliability of each of these thresholds (lactate and ventilatory) to discard methods with high variability because variability reduces our ability to detect statistical differences among tests. Finally, we compare all tests to a proxy measurement of performance (i.e., maximal lactate steady state; MLSS) to study which is a better test to predict endurance performance (i.e., more reliable and valid).

When the two GXT were compared to test reliability, we found that RER = 1, V-Slope and D_MAX_ were the less reliable determinations with higher CV (≥ 5%), lower ICC (< 0.80) and higher Bland-Altman bias (> 5) than the rest of the indexes (Table 2). Specifically, D_MAX_ was the least reliable of all used method, returning CV values above 10%, ICC of 0.57 and Bland-Altman bias above 15. In contrast, VO_2max_, VT_1_ and VT_2_ were the physiological indexes with the highest reliability (Table 2). Other authors have also found high VO_2max_ (CV = 2% and ICC = 0.97 [26]; r = 0.92 [16]) and anaerobic threshold (VT_2_ r = 0.91 [27] reliability in well trained cyclist. To our knowledge, no author has compared the reliability of such an extensive battery of tests, and thus our data allow us to discourage the use RER = 1, V-Slope and D_MAX_ when other indexes are available.

We found that the workloads at the first ventilatory threshold (i.e., VT_1_) could be determined by measuring the workload at which lactate start to increase above resting values (i.e., LT) since there was a high level of agreement between these two measurements (Table 1). The average difference between these two tests (VT_1_ vs. LT) was of only 14 watts which is half of which could be discriminated in each increment of our graded test where workloads increased 25 watts per stage. The coincidence between LT and VT_1_ has been known from the seminal studies of Wasserman and co-workers in the seventies [28]. That agreement between VT_1_ and LT has been confirmed by Lucia and co-workers using elite endurance cyclists [4] and thus our findings are not novel in this regard but rather confirmatory.

In contrast to the situation with VT_1_, there is no clear agreement as to which is the lactate threshold that better reflects VT_2_. In our experiment, VT_2_ statistically agreed with D_MAX_ and RER = 1 (Table 1). However, as discussed above, reliability is low for these two indexes and thus there are not fair substitutes of VT_2_. Out of the reliable indexes, VT_2_ workload coincided with LT+2 mMol·L^-1^ and with the workload that elicits blood lactate concentration of 4 mMol·L^-1^ (i.e., OBLA 4Mm). Lastly, the workload that elicits LT+0.5 mMol·L^-1^ nicely agreed with the maximal workload that can be maintained without elevations in blood lactate concentration (i.e., MLSS; Table 1). Thus, coaches and athletes could, by measuring LT, LT+0.5 and LT+2 mMol·L^-1^ detect the workload at VT_1_, MLSS, VT_2_ and readily advice optimal performance intensity for training or endurance events.

Lactate and ventilatory thresholds are the manifestation of and underlying metabolic events where homeostasis is lost. For instance, VT_1_ (i.e., anaerobic threshold; [28] is the intensity at which ventilation and VCO_2_ increase in parallel. The increase expired CO_2_ is generated by the HCO_3_^-^ buffering of lactic acid that reaches the blood [29]. VT_2_ (i.e., RCP, [28] in turn represents a work intensity at which blood lactate accumulation rises considerable and there is hyperventilation to buffer acidosis (i.e., ventilatory compensation). Thus, VT_2_ represents the highest metabolic rate at which the system is able to maintain an elevated but stable metabolic acidosis. Exercise above these thresholds results in accumulation of fatigue inducing metabolites [30], rapid increases in intramuscular and arterial lactic acid, hydrogen concentration [31] and changes in motor unit recruitment [32]. Several authors have reported that long-term training programs at each of these thresholds or intensity zones will produce particular and different central and peripheral adaptations [33–35].

**Fig 4:**
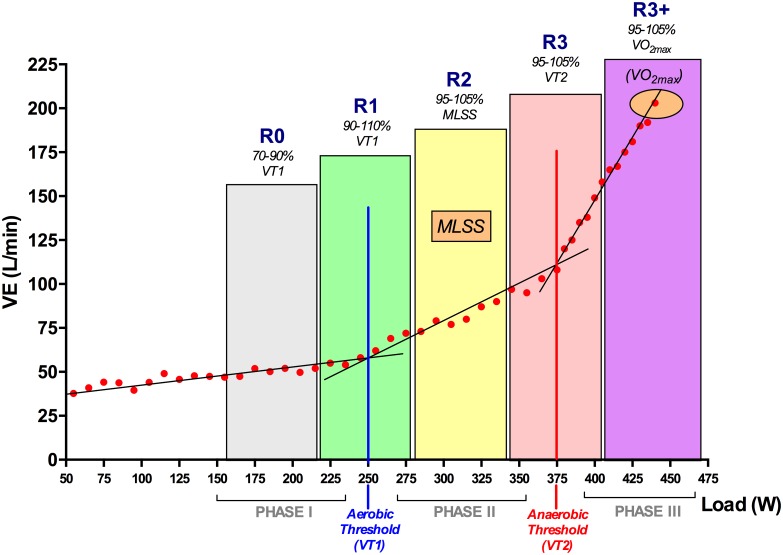
Scheme of physiological events and training zones proposed by the authors.

With the objective of applying our findings to training and competition, we developed Tables 3 and 4. In Table 3 the lactate indexes better associated with VT_1_, MLSS and VT_2_ just defined (i.e., proxy for LT, LT+0.5 and LT+2 mMol · L^-1^, respectively) are presented with their correspondent percent of HR_MAX_, heart rate reserve (HRR) and RPE showing the upper and lower 95% confidence interval. In this way, athletes and coaches that only have access to monitoring heart rate and/or RPE could locate the intensities of VT_1_, MLSS and VT_2_. Furthermore, we proposed several training zones based in a previous publication [35] now locating them with respect to LT, LT+0.5 and LT+2 mMol · L^-1^ (Fig 4). We hope that this will allow athletes and coaches to undergo training at intensities that induce different metabolic adaptations while only needing measurement of HR_MAX_, HRR or RPE (Table 4).

**Table 3:** 95% confidence interval for each physiological event.

	HR_Max_ (%)	HRR (%)	RPE
**VT**_**1**_ **(LT)**	71%—78%	62%—71%	11—12
**MLSS (LT+0.5)**	81%—87%	76%—83%	13—13
**VT**_**2**_ **(LT+2.0)**	87%—92%	83%—89%	14—15

HR_Max_ Maximal heart rate, *HRR* Heart rate reserve, *RPE* rate of perceived exertion.

**Table 4:** Personal author's approach for exercise prescription (training zones).

Percentage	Zone	HR_Max_ (%)	HRR (%)	RPE
**70%—90% VT**_**1**_ **or LT**	R0	52%—67%	53%—62%	8—9
**90%—110% VT**_**1**_ **or LT**	R1	67%—82%	62%—71%	10—11
**90%—100% MLSS or LT+0.5**	R2	82%—87%	71%—85%	12—14
**95%—105% VT**_**2**_ **or LT+2.0**	R3	87%—95%	85%—94%	15—16
**95%—105% VO**_**2max**_	R3+	95%—100%	95%—100%	17—19

HR_Max_ Maximal heart rate, *HRR* Heart rate reserve, *RPE* rate of perceived exertion.

Some studies in the literature present data on both ventilatory and blood lactate thresholds during GXT, although their main objective is not to compare them. Regarding VT_1_, Coyle et al. [22] established LT +1.0 mMol·L^-1^ as the lactate threshold workload that better matches the workload at VT_1_. This study was conducted on patients with ischemic heart disease, which could be behind the difference between our studies. Lucia and co-workers [4] detected a high agreement between VT_1_ and LT (321±8 W vs. 319±10 W) in elite endurance cyclists, and our data corroborates their findings in well trained cyclist. On the other hand, in an attempt to locate the VT_2_ workload through a CBL test, Smekal et al. [36] found that a value of 4.1±1.0 mMol·L^-1^ agrees with VT_2_ in active and healthy men and women, which coincides with our findings (Table 1). Nevertheless, Davis and co-workers [37] suggest that anaerobic thresholds have frequently been determined using blood lactate concentrations of less than 2 mMol·L^-1^ as a reference point. Thus, workload at VT_2_ could be notably underestimated when following these previous reports.

Detection of MLSS intensity is particularly important since a substantial portion of aerobic training in athletes is carried out at MLSS intensities [8,34,35,38,39,40]. Our results indicate that LT + 0.5 mMol·L^-1^ during a GXT is a valid predictor of MLSS workload in well trained cyclist (p = 0.449; r = 0,78; Bias = -4.5). The determination of LT + 0.5 mM·L^-1^ during an incremental exercise test as a proxy of MLSS will reduce testing time and the fatigue associate with the several MLSS trials required to achieve the determination. In agreement with Skinner and McLellan [41], our results showed that MLSS does not correspond to VT_1_ (aerobic threshold) or VT_2_ (anaerobic threshold) but represents an intermediate intensity between both physiological events. This finding is important since numerous authors have proposed that the workload at VT_2_ coincides with the one for MLSS [36,42], making it difficult for coaches and sport scientists to effectively communicate their findings and the effects produced by different training intensities. Other authors have tried to estimate the MLSS workload through CBL detected during a GXT. For example, Beneke [19] found marked differences in the workloads at LT and OBLA_4mM_ in an incremental test with respect to the workload at MLSS in high-level rowers. Recently, Hauser et al. [43], in male trained subjects during a different GXT protocol (40W/4min), found similar evidences to those described in our work. These authors detected differences between the results of LT+1.5 mM·L^-1^ and MLSS (i.e., low validity values). However, contrary to our results, they found great similarities between the OBLA_4mM_ and MLSS values. The discrepancies between studies may be related to our faster increase in workload during the GXT protocol (40 W every 4 min for Hauser et al. [43], while 75 W every 4 min, presently).

To predict performance among a group of competitors and to delimit training zones it is required to assess the workload at the aerobic and anaerobic thresholds. The most accurate way to measure this metabolic event is with the use of indirect calorimetry. Ventilatory thresholds have been shown to accurately track the improvements in endurance performance of elite [4] and well trained endurance cyclist [7]. However, indirect calorimeters are expensive and thus out of reach of many coaches and athletes. Evaluation of anaerobic threshold by CBL is cheaper and often chosen as an alternative method. However, the reliability and validity of anaerobic threshold identification by CBL is controversial. Our data support that capillary blood lactate-based tests are highly reliable and they can be a valid alternative to ventilatory thresholds to identify the workloads at the transition from aerobic to anaerobic metabolism. Furthermore, LT+0.5 mM·L^-1^ is an alternative test highly correlated with MLSS. These correspondences here presented between ventilatory and CBL thresholds, as well as the relationship between them and heart rate and rate of perceive exertion (Tables 3 and 4) apply to our GXT protocol (25W·min^-1^).

## Study limitations

Any other graded (e.g., 25 W· 4 min^-1^) or constant workload protocols, or any other exercise modes (running, swimming or paddling) may change these relationships, and therefore the validity values reported in this work could decline.

## Supporting Information

S1 File Graded exercise test and maximal lactate steady state test results. (XLSX)
